# Use of Echocardiography Under Hypoxic Stress Without Exercise to Assess Right to Left Shunting

**DOI:** 10.3390/jcdd12110435

**Published:** 2025-11-03

**Authors:** Cecilia Villa Etchegoyen, Rachel E. Wraith, Lisa S. Brown, Karen K. Breznak, Rohit Mital, Steven J. Lester, Chadi Ayoub, Said Alsidawi, Justin N. Shipman, Juan M. Farina, Reza Arsanjani, Jan Stepanek

**Affiliations:** 1 Department of Cardiovascular Medicine, Mayo Clinic, Scottsdale, AZ 85054, USA; 2 Aerospace Medicine Program, Department of Internal Medicine, Mayo Clinic, Scottsdale, AZ 85054, USA

**Keywords:** echocardiography, stress test, shunt, high altitude, dyspnea, hypoxic simulation testing

## Abstract

Acute exposure to hypoxia will induce right ventricular (RV) hemodynamic changes and may increase the degree of right-to-left shunting, which can contribute to dyspnea at altitude. In this retrospective study, 125 patients (median age 66 years; 50.4% women) with unexplained dyspnea at altitude underwent hypoxic simulation testing (HST) with transthoracic echocardiography (TTE). During simulated hypoxia (mode (Min-Max) altitude: 8000 (6000–18,000) ft, were observed a significant decrease in oxygen saturation (97% (95–98) vs. 88% (82–92), *p* < 0.001) and RV free wall longitudinal strain (−19.6 ± 3.99% vs. −17.3 ± 4.17%, *p* < 0.01), an increase in RV systolic pressure (RVSP: 26 (23–30.5) vs. 29 (25–36.5) mmHg, *p* < 0.001). No significant changes were observed in TAPSE (20 (18–23) vs. 20 (19–24) mm) or S wave (0.12 (0.11–0.14) vs. 0.13 (0.12–0.14) m/s). Right-to-left shunting was present in 47.2% of patients and 11.9% exhibited inducible shunting only under hypoxia. However, under hypoxia, there were no significant differences in RV hemodynamic parameters or saturation between those with and without shunting. TTE with HST is useful to characterize both cardiopulmonary response and the dynamic changes in right-to-left shunt behavior under hypoxic stress.

## 1. Introduction

Exposing the human body to an environment where oxygen availability is significantly reduced, such as high altitudes, results in hypobaric hypoxia. The fall of oxygen tension triggers a variety of physiological changes in the cardiovascular system that may result in dyspnea. While healthy individuals undergo successful physiological acclimatization to high altitude over a few days, a considerable number of individuals experience exaggerated or prolonged symptoms [1]. Many people are transiently exposed to hypobaric hypoxia during commercial air travel and visits to high-altitude destinations. The aircraft cabin pressurization is equivalent to an in-flight altitude of 5000–8000 ft contingent on aircraft type and flight altitude [2,3], leading to a decrease in the alveolar partial pressure of oxygen (O_2_) compared to sea level.

The acute decrease in arterial O_2_ pressure triggers a series of compensatory physiological responses, including hyperventilation, increased heart rate and pulmonary vasoconstriction, to maintain adequate O_2_ delivery to the tissues [4,5]. However, a subgroup of individuals experience disabling symptoms upon rapid ascent to high altitudes, including dyspnea, fatigue and exercise intolerance [6]. These individuals are characterized by exaggerated arterial hypoxemia and hypocapnia at high altitude, which may initiate a maladaptive response whereby the mechanisms are not fully understood. Symptoms may result from an inefficient cardiopulmonary response, including an excessive elevation in pulmonary vascular resistance and right ventricle (RV) overload, and the exacerbation of shunts, such as patent foramen ovale (PFO) or transpulmonary shunts, which can lead to more severe hypoxemia [7,8,9].

Hypoxic simulation testing (HSTs) simulates changes in physiology that occur at altitude by administering a mixture of gases with a lower O_2_ content to simulate different elevations [10]. This test may allow for detection of unexpected excessive hypoxemia and associated signs and symptoms. The addition of transthoracic echocardiography (TTE) during hypoxic exposure testing can detect right heart abnormalities (increased RV pressures and changes in RV hemodynamics), as well as the presence of right-to-left shunts. However, current evidence on the effects of acute high-altitude simulation on RV hemodynamics has yielded conflicting results, and there are no consistent recommendations for assessing cardiopulmonary response during altitude testing [6]. Recognizing the physiological responses of the RV to acute hypoxia and the behavior of shunts may be important to better understand the mechanisms underlying signs and symptoms at high altitude.

The aim of this study was to assess changes in the RV hemodynamics and the shunt behavior during HSTs with TTE without exercise in patients referred for high-altitude symptoms.

## 2. Materials and Methods

A retrospective observational study of adult (≥18 years old) patients with unexplained dyspnea at high altitude who were referred to the Aerospace Medicine program at a single institution (Mayo Clinic Arizona) between 1 October 2021 and 31 March 2025 was performed. All patients underwent clinical assessment including arterial blood gas analysis and acid base determination prior to hypoxic exposure. Patients with a known history of cardiopulmonary diseases that could explain the presence of high-altitude symptoms were excluded from the study. 

### 2.1. Hypoxic Simulation Testing Without Exercise—Transthoracic Echocardiography

The full protocol of HTSs was described in previous work of the group [11]. Briefly, subjects were given different FiO_2_ concentrations to simulate altitude levels specific to each patient history (from 6000 to 18,000 ft). Vital signs (heart rate, arterial blood pressure, and end-tidal carbon dioxide levels, O_2_ saturation) were monitored during the test via finger pulse-oximetry and both baseline and peak hypoxia echocardiographic measurements were collected [10].

TTEs were performed using standard ultrasound scanners (Philips iE33; Phillips Medical Systems, Andover, MA, USA; GE Vivid E9, GE Healthcare, Milwaukee, WI, USA) and as per American Society of Echocardiography (ASE) guidelines [12,13]. All measurements were performed by a single experienced cardiologist with an American Society of Echocardiography certification for special competency in echocardiography (RA). TTE images were obtained at baseline and during peak hypoxia to evaluate RV hemodynamics, function and possible shunts.

RV systolic pressure was estimated from tricuspid regurgitation jet velocity and right atrial pressure by IVC size and collapsibility. RV systolic function was assessed by TAPSE (M-mode displacement of the lateral tricuspid annulus), S wave (tissue Doppler systolic velocity), and RV free wall longitudinal strain (percentage of systolic shortening from base to apex in the RV-focused 4-chamber view). Strain analysis was performed offline using EchoInsight ZP v5.1 (Epsilon Imaging, Inc., Ann Arbor, MI, USA) or TomTec v2.0 (TomTec Imaging System GmbH, Bavaria, Germany).

To detect any possible shunt, agitated saline testing was performed at baseline and maximum hypoxia according to ASE guidelines. A shunt was considered present if bubbles appeared in the left chambers; classified as intracardiac if seen within three beats and transpulmonary when appearing later. If initial testing was negative, a Valsalva maneuver was performed to transiently increase right atrial pressure during contrast injection both at baseline and under hypoxia.

### 2.2. Statistical Analysis 

Data were presented as means with standard deviations (mean ± SD) or median with 25th and 75th percentiles (median (P_25_–P_75_)) for continuous variables and frequencies and percentages for categorical variables. Statistical comparison between baseline and peak hypoxia results was performed using Student’s paired t-test and Wilcoxon signed- rank test for continuous variables, or McNemar test for categorical variables comparison. Independent groups were compared with Student’s non-paired t-test and Mann–Whitney U test for continuous variables, or Chi-squared test for categorical variables. For all analysis, *p*-values of <0.05 were considered statistically significant. Statistical analyses were conducted using RStudio, version 2025.5.1.513 (Boston, MA, USA).

## 3. Results

The study included 125 patients, with a median age of 66 (50–74) years and 63 (50.4%) females. On baseline TTE, median values were within the normal range including left ventricle ejection fraction (LVEF) 62% (59–65), TAPSE 20 mm (18–23), S wave 12.1 cm/s (11.2–14.1) and RV systolic pressure 26 mmHg (23.0–30.5). The mean value for RV free wall longitudinal strain was −19.6 ± 3.99%. At baseline, no patient showed TTE signs compatible with increased left heart filling pressures, and no significant cardiac valve disease was detected. Furthermore, no significant abnormalities were seen in the remainder of the baseline TTE parameters (Table 1).

The majority of patients (79.3%) received a mixture of gases to simulate an altitude of 8000 ft according to the altitude at which they referred symptoms. Regarding the comparison between baseline and peak hypoxia results, a significant reduction was seen in O_2_ saturation (97% (95–98) vs. 88% (82–92), *p* < 0.001). At peak hypoxia, there was a significant increase in the RV systolic pressure (baseline 26 (23–30.5) mmHg vs. peak hypoxia 29 (25–36.5) mmHg, *p* < 0.001). The RV longitudinal function assessed by TAPSE and S wave showed no significant change under hypoxia, while RV strain was significantly decreased (−19.6 ± 3.99% vs. –17.3 ± 4.17%, *p* < 0.01) (Table 2).

Additionally, right-to-left shunting by TTE at baseline was identified in 57 (45.6%) patients, of whom 4 were detected only with the Valsalva maneuver. Of these, only 13 (25.5%) had a previous diagnosis of PFO and 8 (14%) of an atrial septal defect. Regarding the gradation of the shunt, most of the cases were small (n = 40, 78.4%), with 5 (9.8%) and 6 patients (11.8%) showing moderate or large right-to-left shunts, respectively (Table 2). In 53 (42.4%) patients, the shunt was evident at baseline with no changes at peak hypoxia, while the severity of shunt increased at peak hypoxia in 13 (10.4%) cases, and the shunt was only evident during hypoxia in 9 (7.2%) patients. The Valsalva maneuver at the peak of hypoxia did not unmask any new shunts, although one small shunt increased to moderate with the execution of the maneuver.

There were no statistically significant differences in RV longitudinal strain, O_2_ saturation, or systolic function parameters during hypoxia between those individuals who had shunts versus those who did not (Table 3). Among individuals with right-to-left shunt, 37 (56.1%) reported O_2_ saturation below 88%, while 28 (42.4%) showed abnormal RV strain, however both features were reported in only 15 (22.7%) patients. In the subgroup of patients with increased shunt under hypoxia, 10 (76.9%) had desaturation, 7 (53.8%) had abnormal RV strain and 5 (38.5%) displayed the two characteristics concurrently.

## 4. Discussion

Previous studies by our group demonstrate that the use of TTE in conjunction with HST without exercise effectively identifies right-to-left shunts and changes in RV function and hemodynamics [11]. The current manuscript provides the largest cohort evaluated using this combination of techniques, providing a more in-depth analysis of the implications of the presence of a right-to-left shunt on RV behavior under hypoxia. The present analysis demonstrated a significant increase in RVSP and a significant decrease in RV systolic function as measured by longitudinal free wall deformation in hypoxia, whereas TAPSE and S wave measurements showed no significant changes. In addition, a significant increase in the presence and severity of right-to-left shunts was observed under hypoxia, although individuals with a shunt did not show worse RV performance, a significantly increased RVSP, or greater desaturation than those without abnormal flow. 

There has been conflicting evidence regarding the relationship between the presence of a right-to-left shunt, specifically a PFO, and its role in the development of symptoms at altitude. Some authors report that PFO is significantly more common in mountaineers with altitude sickness, including a five-fold increase in those with high-altitude pulmonary edema (HAPE), and that its presence is associated with an increased risk of altitude sickness independent of age, sex, prophylactic acetazolamide use, or acclimatization status [7,14]. On the other hand, studies involving healthy volunteers (assumed asymptomatic) exposed to normobaric hypoxia equivalent to 15,000 feet for 7 to 10 h found no significant increase in high-altitude symptoms or pulmonary artery pressure in individuals with PFO [15]. It follows from the above that evaluating the degree of significance of the shunt is crucial for comparing and extrapolating results. DiMarco’s cohort consisted of likely asymptomatic individuals with presumed “innocent” or hemodynamically insignificant PFOs [14], whereas our cohort included symptomatic patients with suspected altitude intolerance, in whom PFOs and other shunts may behave differently under hypoxic stress. Therefore, although both studies evaluate right-to-left shunts in hypoxia, they represent different ends of the clinical spectrum and are not directly comparable.

**Figure 1:**
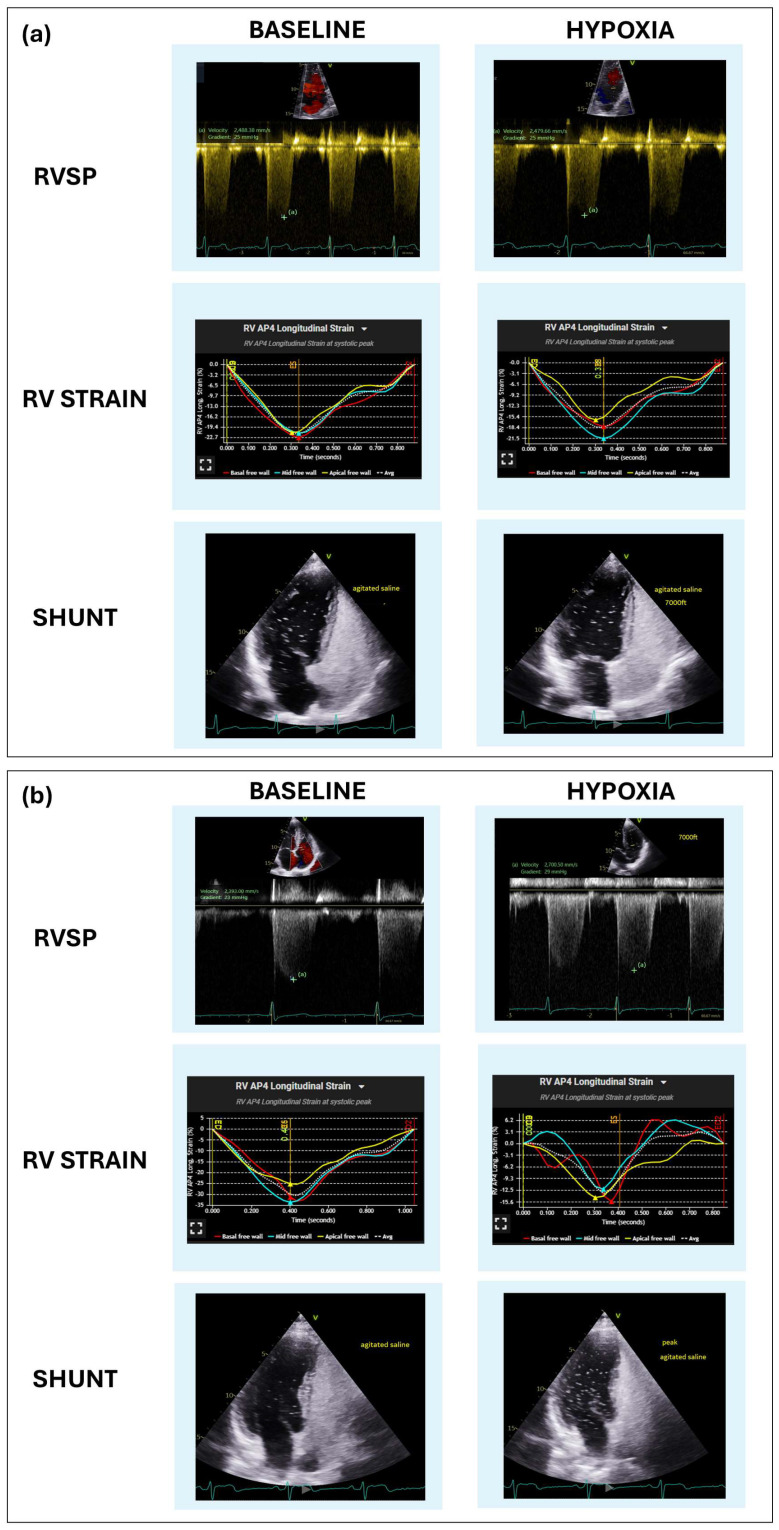
Graphical representation of shunt assessment ("innocent" vs. "guilty") in the hypoxic stress test without exercise and TTE. From top to bottom in panels (**a**) and (**b**), apical 4−chamber view TTE images during the contrast bubble test, results of RV longitudinal strain and tricuspid regurgitation jet for calculating RVSP before and during the peak of hypoxic stress. (**a**) Small shunt, without increased its magnitude and minimal modifications in hemodynamic parameters and oxygen saturation under hypoxia (“innocent” shunt). (**b**) Small shunt that with an increased under hypoxia and significant oxygen desaturation, increase in RVSP and a fall in RV longitudinal strain and (“guilty” shunt).

The current results reported an overall shunt prevalence of 52.8% in our symptomatic population, a higher percentage than the general population [16,17]. In this study, 25.5% of shunts were PFO with a lower incidence of atrial septal defects and intrapulmonary shunts. These findings reflect the etiological heterogeneity of right-to-left shunts and their potential impact on systemic oxygenation [18]. Furthermore, the fact that the percentage and magnitude of right-to-left shunts increased in hypoxia suggests that assessing shunts at rest, or at sea level, is not sufficient for the evaluation of symptomatic patients at high altitude. This highlights the importance of combining dynamic hypoxic testing with TTE to explore the functional significance of right-to-left shunting. In our cohort, more than half of the patients with right-to-left shunt experienced clinically relevant desaturation, 42.4% showed abnormal RV strain, and both alterations were present in 22.7%, suggesting that not all shunts are functionally equivalent. In the subgroup with increased shunt under hypoxia, the prevalence of abnormal responses was even higher, with 76.9% experiencing desaturation, 53.8% with abnormal RV strain, and 38.5% exhibiting both features. These findings support the existence of “innocent” versus “guilty” nature of a right-to- left shunt. In this regard, Figure 1 shows a clear example of two individuals in this cohort with small right-to-left shunts at rest, with and without changes in magnitude under hypoxia, and a very different response in O_2_ saturation and hemodynamic parameters. Panel (a) shows a right-to-left shunt without altered magnitude under hypoxia and slight drop of O_2_ saturation and variations of RV longitudinal strain and RVSP (likely “innocent” PFO). On the other hand, panel (b) shows a significant increase in shunting, a significant decrease in O_2_ saturation and declining RV hemodynamic parameters (representing a “guilty” PFO). Under hypoxic stress the change of magnitude of the right-to-left shunt and its impact on O_2_ saturation and cardiac hemodynamics allows a distinction between functionally significant (“guilty”) versus insignificant (“innocent”) lesions beyond mere static descriptions of anatomical features.

The increase in RVSP suggests that the RV responds rapidly to the decrease in oxygen partial pressure by increasing afterload, mediated by hypoxic pulmonary vasoconstriction. The cardiovascular response to hypoxia results in pulmonary vasoconstriction to redistribute blood flow to better-ventilated areas, however this may also result in RV overload if hypoxia is sustained or severe [19,20]. Although the absolute increase in RVSP observed in this study (from 26 to 29 mmHg) remains within the normal range, this statistically significant change during a short period of induced hypoxia represents an acute physiological response of the pulmonary circulation, which may be clinically relevant in susceptible individuals. In such patients, even small hemodynamic variations, when combined with reduced RV systolic function and increased right-to-left shunting, may contribute to the development of dyspnea or exercise intolerance at altitude, even in the absence of evident structural cardiac pathology at baseline. Moreover, Strange et al. identified a lower threshold for increased mortality risk (RVSP more than 30 mmHg), indicative of pulmonary hypertension, suggesting that even mild elevations in RVSP could have clinical relevance [21].

The finding that TAPSE and S wave parameters remained unchanged, whereas RV strain significantly declined under acute hypoxia, suggests that hypoxic stress may unveil latent right ventricular dysfunction. RV longitudinal strain is well established as a more sensitive and load-independent marker of early systolic impairment compared with conventional indices such as TAPSE and S wave [9]. The latter measures often remain within normal limits until more advanced stages of disease, underscoring the incremental value of strain analysis for the detection of subclinical RV dysfunction. In patients presenting with unexplained dyspnea at altitude, the integration of TTE with HST may therefore facilitate the identification of functional abnormalities not apparent under normoxic conditions.

The absence of a clear difference between groups regarding the presence of right-to-left shunting and impaired RV function or lower O_2_ saturation levels under hypoxia needs to be considered cautiously. The fact that the individuals included in this cohort had baseline preserved RV function and an absence of pulmonary pathology may indicate that the adequate RV compensatory capacity and pulmonary reserve may have partially mitigated the adverse hemodynamic effects of right-to-left shunting. Conversely, the degree of right-to-left shunting observed may not have reached a hemodynamic threshold sufficient to produce clinically significant impairments in ventricular function or systemic oxygenation during these tests. This could be particularly relevant given the limited duration and intensity of the hypoxic stress imposed during the evaluations. These results are consistent with previous studies that have reported that the presence of a right-to-left shunt under basal conditions or under stress may not always be associated with immediate functional impairment but may constitute a risk factor for clinical events, such as paradoxical embolisms or decompensation in situations of additional stress [22]. The high prevalence of right-to-left shunts and their behavior under simulated hypoxia highlight the importance of considering this condition when evaluating patients with unexplained symptoms at altitude or during exposure to environmental hypoxia, such as commercial flights or activities under reduced barometric pressure [9]. Early and accurate detection of shunts, especially through noninvasive techniques such as contrast-enhanced echocardiography and ventricular strain, can facilitate the identification of at-risk individuals and guide prevention strategies [23,24]. Future studies should explore the longitudinal outcome of these patients, assessing whether the transient increase in shunting under hypoxia may be a marker for future clinical events.

This study has limitations that may influence the interpretation of the results. Shunt assessment was performed using transthoracic echocardiography, which, although a useful and accessible tool, may have limited sensitivity in detecting small or transient shunts, especially compared to transesophageal echocardiography (TEE) or more advanced imaging techniques [25]. However, in the detection of right-to-left atrial communication, a strong agreement is reported between TTE with agitated saline injection and TEE (sensitivity 99%, specificity 85%) [26], and TTE with first-generation contrast can be used to detect a PFO [12]. A shortcoming of all echocardiographic shunt assessments lies in the use of the superior vena cava (SVC) territory (injection of bubble contrast into forearm vein) to investigate a shunt (PFO) that emanates from an in utero vestige of a shunt of the inferior vena cava territory to the left side of the heart and not the SVC.

TEE is the gold standard for the anatomical visualization of PFOs [11], offering high spatial resolution and the ability to directly observe the interatrial septum. However, it is semi-invasive procedure and requires sedation, and as such it is not possible to perform the Valsalva maneuver. This may result in underestimation of the shunt due to physiological alterations during the procedure. On the other hand, noninvasive techniques for shunt assessment, such as quantitative shunt assessment with transcranial Doppler (TCD), cardiac magnetic resonance (CMR), and ventilation-perfusion (V/Q) scanning, offer partial information on the characteristics and functional impact of the shunt. TCD is sensitive for detecting right-to-left shunts and allows real-time quantification during physiological maneuvers [27], but it lacks anatomical detail and cannot distinguish intracardiac from intrapulmonary shunts. CMR provides excellent soft tissue characterization and can assess associated cardiac abnormalities, but it has limited temporal resolution for bubble tracking and is not widely used for routine PFO evaluation [28]. Finally, V/Q scanning can detect and quantify extrapulmonary shunts, but it is nonspecific and lacks the spatial resolution necessary to localize the shunt site.

One of the challenges in assessing and investigating right-to-left shunts (most commonly PFO) is the historical reliance on mere anatomical high-risk features (e.g., aneurysmal atrial septum, hypermobile atrial septum, persistent Eustachian valve and Chiari network), resulting in heterogeneity of the literature. High risk anatomy features are important, but do not provide for a dynamic assessment (stress testing) of the pathophysiologic significance of an anatomic finding and hence limits our ability to assign guilt to a PFO [29]. Comorbidities such as sleep apnea (added hypoxic stress with intermittent Valsalva maneuver with gasping or residence at high altitude) should raise clinical suspicion and need for further evaluation and assessment to rule out clinically significant contribution of right-to-left shunts. In addition, body position is known to affect propensity for right-to-left shunting (orthodeoxia/platypnea or platydeoxia/orthopnea) and should be on the mind of the clinician when a shunt is assessed either anatomically or through stress (hypoxia, exercise) to make sure a clinically significant finding is not missed [30]. 

Other limitations of the study include the fact that the simulation of hypoxia with normobaric hypoxia at 8000 ft, although representative of altitude, will not fully reproduce the physiology of hypobaric hypoxia under reduced barometric pressure, where the effects on ventricular function and shunting could be more pronounced. Furthermore, in our study there was a lack of control group of healthy individuals without high-altitude symptoms. Finally, the current lack of long-term clinical follow-up limits the ability to correlate echocardiographic findings with relevant future clinical events, such as episodes of decompensation, paradoxical embolisms, or progressive functional decline. 

Future work is ongoing to combine hypoxic stress with exercise, in an attempt to more fully simulate the physiologic state of individuals at altitude during physical activity, combining the stressor of normobaric hypoxia and exercise, which further increases pulmonary arterial pressure response and is expected to provide even higher diagnostic yield. 

## 5. Conclusions

In patients with dyspnea of unknown cause, the combination of transthoracic echocardiography and simulated hypoxia testing without exercise represents a valuable tool for the dynamic assessment of RV function, pulmonary arterial pressure, and right-to-left shunts. In this cohort, we observed a significant reduction in RV systolic function, an increase in estimated pulmonary artery pressure, and an increase in both the presence and severity of right-to-left shunts during hypoxia, findings that may contribute to the development of desaturation and exertional dyspnea at altitude. These observations support the importance of functional rather than purely anatomical assessment of right-to-left shunts. Specifically, our findings help distinguish “guilty” PFOs, characterized by increased shunt magnitude and associated desaturation under hypoxia, from likely “innocent” PFOs, in which the shunt remains small and does not result in clinically significant oxygen desaturation. Identifying these functionally significant shunts may be critical for understanding symptomatology and guiding potential therapeutic interventions. Future research should consider incorporating both hypoxic and exercise stress, as this combination may better replicate the physiological demands experienced by patients at altitude, particularly among physically active individuals.

## Figures and Tables

**Table 1:** Demographic and baseline echocardiographic characteristics of the overall population and according to detection of right-to-left shunt.

	Overall Population *n* = 125	No Shunt *n* = 68 (54.4%)	Shunt *n* = 57 (45.6%)	*p*-Value
**Age (years)**	66 (50–74)	67.00 (55.0–75.00)	65.00 (48.0–72.00)	0.306
**Sex** Female *n* (%) Male *n* (%)	63 (50.4) 62 (49.6)	34 (50.7%) 33 (49.3%)	27 (49.1%) 28 (50.9%)	0.9
**BMI (kg/m^2^)**	28.8 (26.6–32.4)	28.9 (26.8–32.8)	28.70 (26.6–32.1)	0.668
**Hematocrit (%)**	43.4 ± 5.02	43.1 ± 5.5	43.9 ± 4.5	0.395
**Hb (g/dL)**	14.3 ± 1.8	14.3 ± 1.7	14.4 ± 1.9	0.675
**SBP (mmHg)**	128.5 ± 15.3	129.1 ± 15.9	127.7 ± 14.7	0.597
**DBP (mmHg)**	77.9 ± 10	77.5 ± 9.4	78.4 ± 10.7	0.872
**HR (bpm)**	70 ± 11.9	70.4 ± 12.4	70.2 ± 11.3	0.919
**Rhythm** Sinus Rhythm *n* (%) Non-Sinus Rhythm *n* (%) Pacemaker *n* (%) Atrial fibrillation *n* (%)	77 (61.6) 43 (34.4) 2 (1.6) 3 (2.4)	40 (58.8%) 25 (36.8%) - 3 (4.4%)	37 (64.9%) 18 (31.6%) 2 (3.5%) -	0.149
**TTE baseline parameters**				
**LVEF (%)**	62.0 (59.0–65.0)	62.0 (59.0–65.0)	62.00 (59.0–65.0)	0.661
**LA Volume Index (mL/m^2^)**	25.6 ± 9.8	24.6 ± 9.1	26.8 ± 10.5	0.347
**LVIVS (mm)**	9.8 ± 1.6	9.8 ± 1.7	9.9 ± 1.4	0.749
**LVPW (mm)**	9.3 ± 1.3	9.5 ± 1.3	9.4 ± 1.1	0.692
**LVEDV (mL)**	92.2 ± 26.8	88.7 ± 22.3	94.9 ± 30.4	0.228
**LVESV (mL)**	36.2 ± 12.2	35.2 ± 10.8	37.1 ± 13.8	0.413
**E-Wave (m/s)**	0.6 ± 0.17	0.66 ± 0.2	0.61 ± 0.2	0.117
**A-Wave (m/s)**	0.62 ± 0.2	0.63 ± 0.2	0.63 ± 0.2	0.944
**E/A Ratio**	1.1 ± 0.5	1.1 ± 0.5	1.1 ± 0.4	0.304
**E/e’ medial**	8.0 (6.3–10.0)	8.33 (6.7–11.1)	7.69 (6.3–8.7)	0.0.08
**E/e’ lateral**	6.2 (5–7.9)	6.5 (5.0–8.6)	5.8 (5.0–7.1)	0.042
**RVSP (mmHg)**	26.0 (23–30.5)	27.0 (23.0–32.0)	26.0 (23.0–28.0)	0.169
**TAPSE**	20.0 (18.0–23.0)	20.0 (18.0–23.0)	20.5 (19.0–23.0)	0.126
**S wave**	0.12 (0.11–0.14)	0.13 (0.10–0.14)	0.12 (0.10–0.14)	0.360
**RV strain**	−19.6 ± 3.9	−19.4 ± 4.2	−19.9 ± 3.8	0.525
**HSTs Target altitude**				
**Target Altitude, ft (mean)**	8236.8 ± 1252.5	8260.29 ± 1479.2	8208.77 ± 924.3	0.813
**Target Altitude, *n* (%)** More than 8000 ft 8000 ft Less than 8000 ft	15 (12.0%) 100 (80.0%) 10 (8.0%)	6 (8.8%) 55 (80.9%) 7 (10.3%)	9 (15.8%) 45 (78.9%) 3 (5.3%)	0.325

Data are expressed as *n* (%), mean ± SD and median (P25–P75) according to distribution. BMI: Body Mass Index, Hb: hemoglobin, SBP: systolic blood pressure, DBP: diastolic blood pressure, HR: heart rate, TTE: Trans-Thoracic Echocardiography, LVIVS: Left Ventricular Inter-Ventricular Septum, LVPW: Left Ventricular Posterior Wall, LVEDV: Left Ventricular End Diastolic Volume, LVESV: Left Ventricular End Systolic Volume, TAPSE: Tricuspid Annular Plane Systolic Excursion, S wave: Systolic Velocity, RVSP: Right Ventricular Systolic Pressure, HST: Hypoxic Simulation Test.

**Table 2:** Comparison between baseline and peak hypoxia outcomes.

	Baseline *n* = 125	Hypoxia *n* = 125	*p*-Value
**Sat O_2_ (%)**	97 (95–98)	88 (82–92)	<0.001
**RVSP (mmHg)**	26 (23–30.5)	29 (25–36.5)	<0.001
**TAPSE (mm)**	20 (18–23)	20 (19–24)	0.7
**S wave (cm/s)**	0.12 (0.11–0.14)	0.13 (0.12–0.14)	0.2
**RV strain (%)**	−19.6 ± 3.99	−17.37 ± 4.17	<0.01
**Shunt presence**	57 (45.6%)	66 (52.8%)	<0.01
**Shunt grade**			
**Intrapulmonary** **Intracardiac** Small Moderate Large	6 (10.5%) 51 (89.5%) 40 (78.4%) 5 (9.8%) 6 (11.8%)	7 (12.3%) 59 (89.4%) 46 (78%) 7 (11.8%) 6 (10.2%)	<0.01

Data are expressed as *n* (%), mean ± SD and median (P25–P75) according to distribution. Sat O_2_: arterial oxygen saturation, RVSP: right ventricular systolic pressure, TAPSE: tricuspid annular plane systolic excursion, S wave: systolic velocity of the tricuspid annulus, RV strain: right ventricle longitudinal strain.

**Table 3:** Comparison of results under hypoxic conditions between the presence or absence of shunt.

	Hypoxia		
	No Shunt *n* = 59 (47.2%)	Shunt * *n* = 66 (52.8%)	*p*-Value
**Sat O_2_ (%)**	89 (83–93)	87 (81–91)	0.177
**RVSP (mmHg)**	30 (24–39)	29 (26–34)	0.860
**TAPSE (mm)**	20 (18–22)	21 (19–24)	0.159
**S wave (cm/s)**	13.1 (11–14.2)	13.2 (12.4–15.1)	0.302
**RV strain (%)**	−17.25 ± 3.6	−17.48 ± 4.62	0.672

Data are expressed as *n* (%), mean ± SD and median (P25–P75) according to distribution. Sat O_2_: arterial oxygen saturation, RVSP: Right Ventricular Systolic Pressure, TAPSE: Tricuspid Annular Plane Systolic Excursion, S wave: systolic velocity of the tricuspid annulus, RV strain: right ventricle longitudinal strain. * Same or increased shunt.

## Data Availability

The data are available from the corresponding author on reasonable request.

## Abbreviations

The following abbreviations are used in this manuscript:

RV	Right ventricle
HST	Hypoxic simulation testing
TTE	Transthoracic echocardiography
RVSP	Right ventricular systolic pressure
TAPSE	Tricuspid annular plane systolic excursion
PFO	Patent foramen ovale
FiO_2_	Fraction of inhaled oxygen
S wave	Lateral tricuspid annulus during systole
ASE	American Society of Echocardiography
SD	Standard deviations
P_25_-P_75_	The 25th and 75th percentiles
LVEF	Left ventricle ejection fraction
HAPE	High-altitude pulmonary edema
TEE	transesophageal echocardiography
TCD	transcranial Doppler
CMR	cardiac magnetic resonance
V/Q	ventilation-perfusion
SVC	Superior vena cava
